# The chemical identity, state and structure of catalytically active centers during the electrochemical CO_2_ reduction on porous Fe–nitrogen–carbon (Fe–N–C) materials†
†Electronic supplementary information (ESI) available: Additional information on the secondary nitrogen precursors used as well as addition al XPS and EXAFS data is included. See DOI: 10.1039/c8sc00491a


**DOI:** 10.1039/c8sc00491a

**Published:** 2018-05-03

**Authors:** Nathaniel Leonard, Wen Ju, Ilya Sinev, Julian Steinberg, Fang Luo, Ana Sofia Varela, Beatriz Roldan Cuenya, Peter Strasser

**Affiliations:** a The Electrochemical Energy, Catalysis, and Materials Science Laboratory , Department of Chemistry , Chemical Engineering Division , Technical University Berlin , Berlin , Germany . Email: pstrasser@tu-berlin.de; b Department of Physics , Ruhr Universität Bochum , Bochum , Germany; c Institute of Chemistry , National Autonomous University of Mexico , Mexico City , Mexico; d Department of Interface Science , Fritz-Haber Institute of the Max Planck Society , Berlin , Germany . Email: roldan@fhi-berlin.mpg.de

## Abstract

We report novel structure–activity relationships and explore the chemical state and structure of catalytically active sites under *operando* conditions during the electrochemical CO_2_ reduction reaction (CO_2_RR) catalyzed by a series of porous iron–nitrogen–carbon (FeNC) catalysts. The FeNC catalysts were synthesized from different nitrogen precursors and, as a result of this, exhibited quite distinct physical properties, such as BET surface areas and distinct chemical N-functionalities in varying ratios. The chemical diversity of the FeNC catalysts was harnessed to set up correlations between the catalytic CO_2_RR activity and their chemical nitrogen-functionalities, which provided a deeper understanding between catalyst chemistry and function. XPS measurements revealed a dominant role of porphyrin-like Fe–N_*x*_ motifs and pyridinic nitrogen species in catalyzing the overall reaction process. *Operando* EXAFS measurements revealed an unexpected change in the Fe oxidation state and associated coordination from Fe^2+^ to Fe^1+^. This redox change coincides with the onset of catalytic CH_4_ production around –0.9 V_RHE_. The ability of the solid state coordinative Fe^1+^–N_*x*_ moiety to form hydrocarbons from CO_2_ is remarkable, as it represents the solid-state analogue to molecular Fe^1+^ coordination compounds with the same catalytic capability under homogeneous catalytic environments. This finding highlights a conceptual bridge between heterogeneous and homogenous catalysis and contributes significantly to our fundamental understanding of the FeNC catalyst function in the CO_2_RR.

## Abstract 2

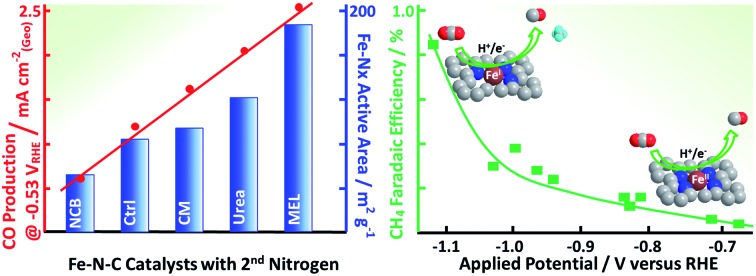
We report novel structure–activity relationships and explore the chemical state and structure of catalytically active sites under *operando* conditions during the electrochemical CO_2_ reduction reaction (CO_2_RR) catalyzed by a series of porous iron–nitrogen–carbon (FeNC) catalysts.

## Introduction

The direct electrochemical conversion of CO_2_ to chemicals and fuels represents an important option to make use of off-gas emissions. Utilization of off-gas CO_2_ helps decrease the consumption of fossil fuels by creating carbon neutral process loops, and thus helps reduce the amount of CO_2_ released in the atmosphere.^1^,^2^ While there are many strategies to reduce CO_2_ emissions, the low-temperature electrochemical conversion comprises a variety of individual processes to recycle CO_2_ streams into valuable chemicals and fuels by electrolysis at room temperature. In these processes, CO_2_ is reduced into more oxygen-poor and thus valuable products.^3^–^6^ Among the possible products from the carbon dioxide reduction reaction (CO_2_RR) are carbon monoxide, formic acid, hydrocarbons as well as alcohols, the liquid carbon fuels.^7^ In the present work we will focus primarily on CO production, although we will also make some comments on these catalysts for methane formation. Historically, CO_2_RR catalysts for CO production were precious metal electrodes, primarily silver and gold.^2^,^6^,^8^ Although some success was achieved with zinc, it had generally lower selectivity to CO.^6^ Current state-of-the-art CO generating catalysts are still based on precious metals, specifically nanoporous and highly defective plasma-treated silver.^2^,^9^,^10^ This state of the art will require further improvement to reach production levels appropriate for commercialization. Additionally, the cost of these precious-metal catalysts is a concern, but recent progress has been made in non-precious metal CO_2_ catalysts. Asadi *et al.* used transition metal dichalcogenides for CO_2_RR in *ionic liquids* reaching faradaic efficiencies of 90% and current densities over 300 mA cm^–2^.^11^ Transition metals incorporated into nitrogen-doped carbon frameworks are also an option for non-precious metal CO_2_RR catalysis. Varela *et al.* introduced single-site solid-state, non-metallic (non-precious) metal–nitrogen–carbon (MNC) materials as a viable catalyst for CO_2_RR to CO even in *aqueous* media.^12^,^13^ They showed very high activities combined with CO production yields and CO faradaic selectivities exceeding Au and Ag catalysts.^12^ Needless to say that the solid-state single-site MNC catalysts offer a tremendous cost advantage over the state-of-art Ag and Au catalysts.

While the investigation of the CO_2_RR on solid-state single-site MNC catalysts is a new and emerging research direction, the CO_2_RR has been extensively studied on molecular porphyrin-containing complexes. The complexing of transition metals in nitrogen-containing frameworks for CO_2_RR dates back to the 70 s, when Meshitsuka *et al.* observed CO_2_RR over Co phthalocyanine.^14^ In 1980, Fisher and Eisenberg measured CO current efficiencies of over 50% on various Ni and Co macrocycles.^15^ More recently, Zhang *et al.* have measured CO efficiencies of 90% and partial current densities of around 9 mA cm^–2^ on Co phthalocyanine supported on carbon nanotubes.^16^ Although these macrocycles do not contain precious metals, it cannot be said that they are cheap. An important development step was the production of similar materials by the heat treatment of separate metal and nitrogen precursors.^17^ Initially developed for oxygen reduction, these metal–nitrogen–carbon (MNC) catalysts provide a less expensive alternative to metal macrocycles.^12^,^17^–^23^ The present work builds on these earth-abundant MNC catalysts by improving performance and increasing the scientific community's understanding of these active and stable catalytic materials.

Many aspects of MNC catalysts have already been discussed in the literature. Often this discussion has centered around the MNC catalysts for the oxygen reduction reaction (ORR), and one of the more contentious debates has concerned the identification of the active sites. Active site identification has been a particularly problematic subject in the literature due to the high porosity and diverse surface chemistry of MNC catalysts. For ORR, a number of active sites have been postulated including pyridinic nitrogen,^24^–^33^ metal centers coordinated with nitrogen atoms,^13^,^24^–^27^,^34^–^36^ and metallic particles protected by layers of carbon.^37^,^38^ Although much of the work has concerned ORR, we can still adopted some ideas from the literature of the possible validity of these active sites for CO_2_RR. With reference to the first suspect, pyridinic nitrogen, Wu *et al.* showed CO_2_RR on various nitrogen-doped carbon foams leading to an increased CO production with increasing pyridinic nitrogen content. Additionally, their DFT calculations indicated that pyridinic species could be highly active sites for CO_2_RR.^39^ Considering metal center sites, a variety of nitrogen coordinations have been postulated for ORR, from relatively simple MeN_2_ or MeN_4_ sites, to double metal centers such as Me_2_N_5_.^34^,^35^ The evidence for these sites' activities towards CO_2_RR is twofold. First, the work on metal macrocycles mentioned previously would indicate that these similar MeN_*x*_ structures should also be active.^14^,^15^ In a recent study done by Ju and Bagger, the Metal–N_4_ motif was proposed as the active site for the CO_2_RR process.^40^ The second piece of evidence is the higher activity of metal-containing catalysts in comparison to metal free.^12^ The last active site possibility, carbon encapsulated nanoparticles, has been investigated for oxygen reduction reaction.^37^,^38^ With regard to CO_2_RR, the metallic content has been thought to be primarily involved in the competing H_2_ generation.^41^,^42^ Thus, achieving high M–N_*x*_ active site density is still challenging by simply using excessive metal precursors, since this rather enhances the formation of metallic/inorganic particles, primarily producing the unwanted H_2_.^41^

In the present work, a number of FeNC catalysts will be explored that were prepared by polymerizing aniline around a carbon support in the presence of an iron salt.^12^,^19^–^22^ The heat treatment and subsequent acid washing to remove the excess metal content result in an active CO_2_RR catalyst.^12^ However, here we consider FeNC catalysts made in the presence of a variety of additional nitrogen precursors, in part to create a diversity of surface chemistries, in part to control the surface active site such as M–N_*x*_ and pyridinic N. These secondary chemical N-sources make the catalysts not only catalytically more active, but they allow a variation of the physico-chemical properties of the resulting FeNC materials. Thanks to their varying surface chemistries we uncover previously unexplored CO_2_RR activity–property relationships and offer new insight in the identity of the active catalyst structure.^18^,^19^ Particular emphasis is given to the chemical state and the structure of the catalytic Fe center during the CO_2_RR catalysis. To get this information, we apply *operando* X-ray absorption spectroscopy and uncover an unusual, yet catalytically very selective Fe(i) oxidation state, which is probably responsible for the formation of CH_4_.

## Experimental methods

### Catalyst synthesis

Ketjen EC 600JD (AzkoNobel) was stirred in 0.5 M HCl for 24 hours and vacuum filtered with DI water to neutral pH. The washed carbon was refluxed in HNO_3_ for 8 hours at 90 °C and again vacuum filtered with DI water to neutral pH. The material at this point will be referred to as pretreated carbon.

For the synthesis, 3 ml of aniline are added into 0.5 liter of 1 M HCl along with 5 g FeCl_3_ and 5 g ammonium persulfate. At this point a secondary nitrogen precursor was added if desired. For the control catalyst (CTRL) no secondary nitrogen precursor was added. The quantity of the secondary precursor is calculated to add 0.333 moles of nitrogen. Various secondary nitrogen precursors were chosen to represent common nitrogen precursors^19^,^43^–^50^ with varying size and nitrogen contents as summarized in Table S1 in the ESI.† This resulted in 7 g melamine (MEL) or cyanamide (CM), 10 g urea (UREA), or 23.6 g nicarbazin (NCB). After one hour of stirring, 0.4 g of pretreated carbon was added. This pretreated carbon has been ultrasonically dispersed in 50 ml of DI water. The resulting mixture was stirred for 48 hours and then dried. After drying, the mixture was ball-milled and heat treated with a ramp of 30 °C per minute to 900 °C and kept at this temperature for one hour in a nitrogen atmosphere. After heat treatment, the material was refluxed in 2 M H_2_SO_4_ overnight and rinsed to neutral pH *via* vacuum filtration. After this acid wash, a second identical heat treatment was performed. At least a second acid wash and third heat treatment were performed on each sample. After this third heat treatment, XRD was used to determine whether the sample had been cleaned of excess residual Fe (usually in the form of FeS). If the sample is not clean, a third acid wash and fourth heat treatments were performed (this was the case for CTRL and NCB). These materials have also been explored as oxygen reduction catalysts in a related paper.

### Physical characterization

Powder X-ray diffraction (PXRD) patterns were recorded on a Bruker D8 Advance instrument with Cu Kα radiation (*λ* = 1.54056 Å) in the 2*θ* range of 10–90° (Fig. S1†). The morphologies of the catalysts were investigated with a scanning electron microscope (SEM, JEOL 7401F, images presented in Fig. S2†). Specific surface area was obtained from N_2_ physisorption measurements conducted on an Autosorb-1 (Quantachome Instruments) using Brunauer–Emmett–Teller (BET) theory. The pore size distributions were calculated from a nonlocal density functional theorem (NLDFT) pore model based on carbon pores with both slit and cylindrical geometries (Fig. S3†). Bulk iron content was measured by Inductively Coupled Plasma (ICP) and Elemental Analysis (EA) was used for the determination of bulk nitrogen and sulfur contents.

### XPS characterization

X-ray photoelectron spectroscopy (XPS) was measured in an ultrahigh vacuum (UHV) system equipped with a monochromatic Al Kα source (*hν* = 1486.5 eV) operated at 14.5 kV and 300 W, and Phoibos 150 (SPECS GmbH) analyzer. For each sample a survey and high-resolution C 1s, O 1s, N 1s, Fe 2p and S 2p regions were measured. The C 1s signal of graphitic-like carbon was used for binding energy calibration and assigned to 285 eV. The Casa XPS software with pseudo-Voight Gaussian–Lorentzian product functions and Shirley background was used for peak deconvolution. Atomic ratios were calculated from XPS intensities corrected by the corresponding sensitivity factors provided by the manufacturing company (SPECS).

### CO chemisorption

CO chemisorption measurements were carried out to quantify the FeN_*x*_ sites per mass of the as prepared Fe–N–C catalysts (Thermo Scientific TPD/R/O 1110). Each experiment was performed on 100–150 mg of the as prepared catalyst in helium condition (He flow: 20 ccm). As a cleaning pretreatment step, the sample was heated up to 600 °C and kept for 15 min. After the sample was cooled down to –80 °C, 6 times CO pulses were carried out to perform the CO chemisorption. The CO-uptake mole amount per gram of catalyst obtained from this measurement could be utilized to evaluate the FeN_*x*_ site density (μmol g^–1^) of each Fe–N–C catalyst. Interestingly, this provides a linear correlation with the interfacial FeN_*x*_ area (m^2^ g^–1^) calculated from BET surface area (m^2^ g^–1^) and FeN_*x*_ site mole fraction (%) obtained from XPS spectra (see eqn (1) and Fig. S4†).

### XAFS characterization

*Ex situ* X-ray absorption fine-structure (XAFS) spectroscopy data were acquired at the undulator beamline P65 of PETRA III storage ring (DESY, Hamburg, Germany) operating at 6 GeV in top-up mode. The experiments were carried out in transmission mode at the Fe K absorption edge (7112 eV). *Operando* XAFS measurements were performed in fluorescence mode at the SAMBA beamline of the SOLEIL synchrotron (Saint-Aubin, France) using a 35-element solid-state Ge detector. A home-built *operando* electrochemical cell was used, with Pt gauze (MaTeck) serving as a counter electrode and a leak-free Ag/AgCl reference electrode (Innovative Instruments Inc, shown in Fig S5†). CO_2_-saturated KHCO_3_ was employed as electrolyte. Raw data reduction was performed using the program Athena.^51^ Analysis of extended X-ray absorption fine structure (EXAFS) spectra was conducted in Artemis by using the FEFF8 code to extract the coordination numbers (CN), interatomic distances (*r*), disorder parameters (Debye–Waller factor, *σ*^2^), and edge energy shift Δ*E*.^52^

### Electrochemical characterization

For electrochemical characterization, an ink was produced containing 15 mg catalyst, 150 μl isopropanol, 800 μl deionized water, and 50 μl 5 wt% Nafion perfluorinated resin solution (Sigma Aldrich). The ink was sonicated for 8 minutes using an ultrasonic horn, and 50 μl of ink were drop cast onto a 1 cm^2^ glassy carbon electrode resulting in a loading of 0.75 mg cm^–2^.

The resulting electrode was inserted into a CO_2_-saturated, 0.1 M KHCO_3_ solution. The electrochemical reduction of CO_2_ was carried out in a two compartment cell divided by a polymer electrolyte membrane (NR212). The electrochemical data were acquired using a SP-200 potentiostat (Biologic). Cyclic voltammetry was firstly carried out on the Fe–N–C catalysts in CO_2_-saturated 0.1 M KHCO_3_ at various scan rates (20 mV s^–1^, 15 mV s^–1^, 10 mV s^–1^, 5 mV s^–1^ and 1 mV s^–1^) to estimate the double layer (DL) capacitance, which is usually thought to be proportional to the electrochemical surface area (ECSA). The potential cycling was performed between –0.1 and 0.42 V_RHE_ to avoid the faradaic process (Fig. S6†). By extracting the double layer current densities at +0.16 V *vs.* RHE (middle of the *E* scanning range), we were able to quantify the double layer capacitance of each catalyst under electrochemical conditions and found that it shows a perfect agreement with the BET surface area, indicating that the BETSA and ECSA are corresponding with each other (Fig. S7†).

For a CO_2_RR measurement, the potential is scanned at a rate of 5 mV s^–1^ from 0.05 *vs.* RHE to the working potential (between –0.5 V and –1.0 V) and then kept at this value for 60 minutes. The sample of the gas was analyzed after 15 and 60 minutes of constant potential with a Gas Chromatograph (Shimadzu GC 2014, Detectors: FID & TCD). Liquid products were measured *via* HPLC (Agilent 1200, Detector: RID) and a liquid GC (Shimadzu 2010 plus, Detector: FID). All potentials are corrected for ohmic resistances unless otherwise noted.

## Results and discussion

**Fig. 1:**
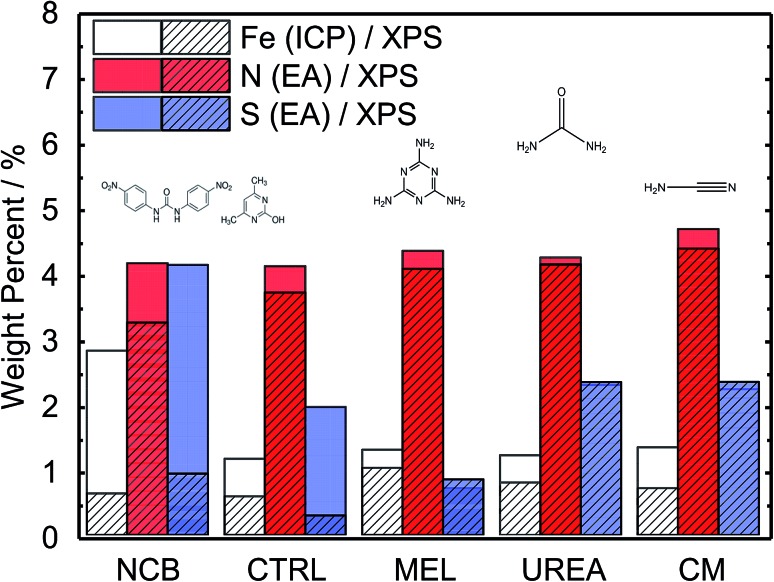
Comparison of Fe–N–C catalysts based on different secondary nitrogen precursors showing bulk iron, nitrogen, and sulfur content as measured by ICP and Elemental Analysis and surface content (*ca.* 2–3 nm) as measured by XPS. Catalysts ordered by increasing surface nitrogen content (XPS).

A range of MNC catalysts were synthesized by varying the secondary nitrogen precursor while keeping the iron, carbon, and primary nitrogen precursor (polyaniline) the same. The secondary nitrogen precursors investigated were melamine (MEL), cyanamide (CM), urea (UREA), and nicarbazin (NCB). These secondary nitrogen precursors cause slight differences in chemistry and morphology. The basic chemical compositions can be seen in Fig. 1, with a comparison of bulk (EA and ICP) and surface (XPS) measurements. By comparing the bulk (open) and surface (hashed) bars, the surface chemistry can be contrasted with that of the bulk. In general, catalysts tend to be surface sparse in Fe, N, and S. This suggests that the surface is rich in carbon. The only exceptions to this observation are the sulfur content of MEL, UREA, and CM. This difference suggests significant sulfur surface functionalization for these catalysts. It is also interesting that these catalysts also contain the highest surface nitrogen contents. For the other two catalysts (NCB and CTRL), the large discrepancies between bulk and surface iron and sulfur contents are ascribed to FeS that can be detected even after an additional acid wash by XRD (Fig. S1†). These particles cannot be easily washed because they lie below the catalyst surface. The small, bulk/surface discrepancy regarding the nitrogen content may also be attributed to similar iron nitride particles below the catalyst surface. Analysis of S 2p spectra (Fig. S8†) however shows three doublets at 164.1, 166.5 and 167.7 eV (for 2p_3/2_), which can be assigned to thiol,^53^ sulfoxide,^54^ and sulfone^55^ species correspondingly, indicating thus exclusive presence of organic sulfur on the surface formed during acid washing.

**Fig. 2:**
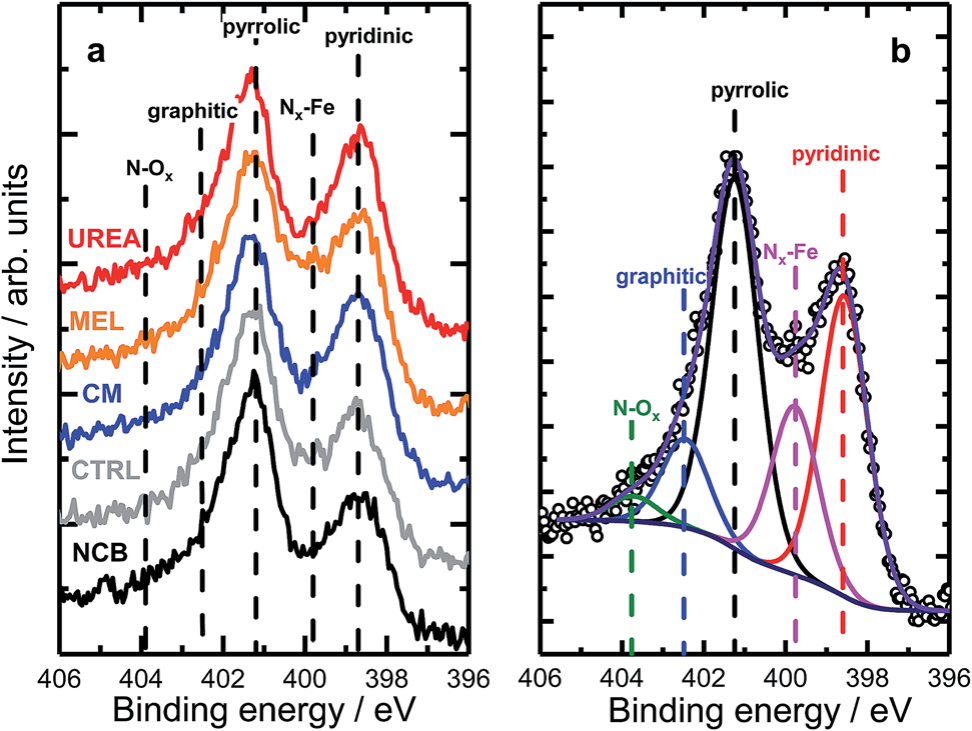
(a) High resolution N 1s XPS data of PANI samples with different N precursors. (b) Example of the deconvolution of a N 1s spectrum acquired for the MEL sample.

Nitrogen 1s XPS region scans are shown in Fig. 2a. All samples show a similar structure with two dominating peaks at 398.7 and 401.3 eV, indicating prevalence of pyridinic and pyrrolic nitrogen in the structure. A spectral valley between those peaks, where N_*x*_–Fe species are reported,^25^ is shallower in the PANI-MEL sample, pointing to a higher concentration of Fe–porphyrin moieties in those samples. Indeed, a more detailed analysis of the N 1s regions, exemplified by the PANI MEL sample in Fig. 2b is summarized in Table 1. The N 1s spectra of Fe–protoporphyrin from Sigma-Aldrich is shown in Fig. S9.† Fe 2p spectra of all PANI samples show a similar structure with Fe 2p_3/2_ having an intense peak centered at 711 eV and a weak satellite observed around 715.5 eV (Fig. S10†). The structure detected is similar to the shape of Fe 2p_3/2_ previously reported for ferrous oxide (FeO).^56^,^57^ It is noteworthy that there are no hints of Fe–N moieties reported at 708 eV (Fig. S11†).^58^ Altogether, the analysis of the Fe 2p_3/2_ spectra indicates that the most iron seen by XPS is in the form of oxidic Fe(ii), while Fe–N_*x*_ species, probed indirectly in N 1s spectra (Fig. 2b and S9), must be lying either in the deeper layers or in pores, not accessible to XPS at Fe 2p due to the lower value of the corresponding inelastic mean free path of photoelectrons.^59^

**Table 1:** Distribution of nitrogen species (in at%) in PANI samples as seen from N 1s XPS spectra deconvolution

Sample	Pyridinic	N_*x*_–Fe	Pyrrolic	Graphitic	N–O_*x*_
NCB	25.0	12.8	44.1	13.0	5.1
CTRL	28.4	12.6	45.9	10.5	2.6
CM	33.8	12.7	42.1	8.6	2.8
MEL	31.8	17.7	36.9	10.1	3.5
UREA	32.7	12.4	40.3	10.7	3.9
Fe–PP (Sigma-Aldrich)	—	100	—	—	—

**Fig. 3:**
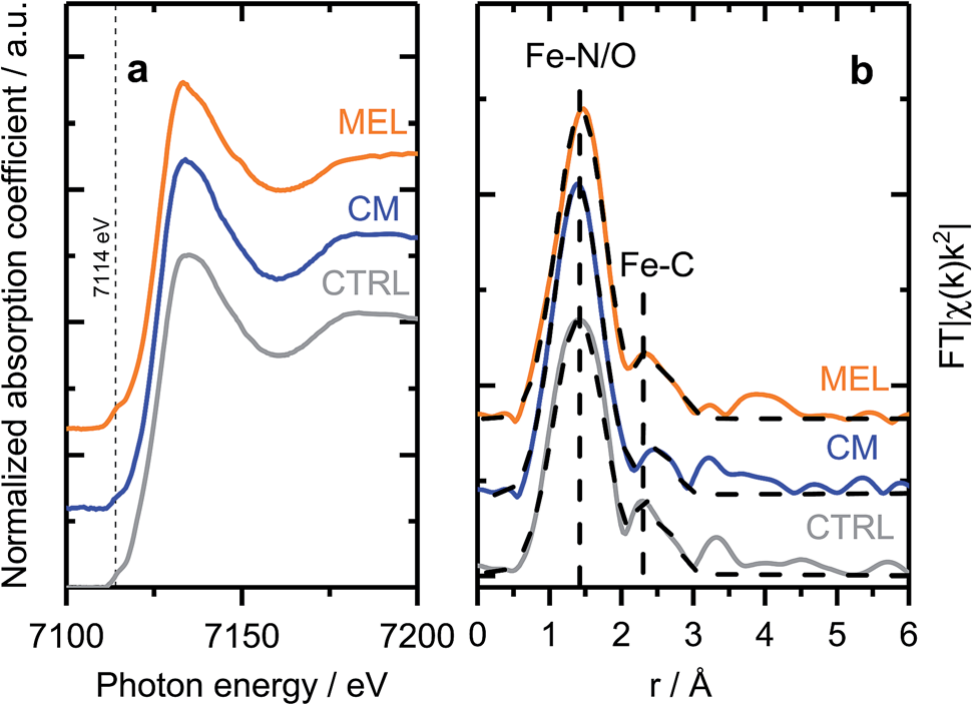
(a) Fe K-edge XANES and (b) EXAFS spectra of selected Fe–PANI samples, dotted lines in (b) show fitted models.

As discussed previously, the discrepancy between surface and bulk iron contents (detected by ICP and XPS as shown in Fig. 1) indicates the presence of iron species not seen by surface sensitive techniques, *e.g.* covered by a carbon layer or isolated in pores of the support. To investigate the nature of those species, X-ray absorption spectroscopy (XAFS) measurements were carried out *ex situ*. X-ray absorption near edge structure (XANES) spectra of selected samples (Fig. 3a) indicate similar chemical state and coordination of iron. The spectra show a pre-edge feature at *ca.* 7114 eV, corresponding to a 1s → 3d electronic transition typical for Fe^3+^ in an octahedral local environment. An intense feature above the absorption edge, between 7126 and 7159 V (so-called white line) has however no similarities with the most common iron oxides (see Fig. S12† for comparison), but is well in line with the results published for similar materials.^60^,^61^ The EXAFS spectra plotted on Fig. 3b (Fe K-edge *k*2-weighted EXAFS data of Fe–PANI samples in *k*-space and analysis as exemplified by CM sample are shown in Fig. S13 and 14†), despite looking somewhat alike, have distinct differences in both, peak positions and intensities. The first peak, originating from a light backscatterer, is observed at 1.46 Å (uncorrected for a phase shift) in the PANI-MEL sample and shifts towards shorter distances in PANI(CTRL) and PANI-CM, 1.42 and 1.39 Å (uncorrected) correspondingly. At the same time, the peak intensity is similar in PANI-MEL and PANI-CM samples, while the PANI sample shows a considerably smaller peak. The second backscattering feature between *ca.* 2.0 and 3.0 Å (uncorrected) is worth special attention. Its location is somehow similar to Fe–Fe backscattering in both common iron oxides with bcc structure (Fig. S12b†), although neither matches exactly in peak position. Zitolo *et al.* assigned a similar structure to crystalline Fe_2_N, formed during pyrolysis in NH_3_.^60^ In our case, formation of nitrides was not observed by any other method. To obtain further details on the local Fe environment, the EXAFS spectra were fitted using an Fe–porphyrin structure^62^ as model and the results are summarized in Table 2. It is seen that the first coordination shell around Fe can be well described by the FeN_4_ moiety. Slight deviations from 4-fold coordination are explained by the interference with iron oxide species which were detected by our surface sensitive XPS method and should be present in the as prepared *ex situ* measured samples. The second next neighbor peak observed at *ca.* 2.4–2.5 Å (uncorrected) is indeed well described by carbon from a porphyrin structure with the real bond distance close to the reference of 3.0 Å. The corresponding coordination number however is significantly lower than 8 in crystalline porphyrin, indicating a highly disordered structure. The latter is also supported by the substantially higher Debye–Waller factors obtained for the PANI samples as compared to the reference iron protoporphyrin sample.

**Table 2:** Best-fit parameters for the Fe K-edge EXAFS spectra of the Fe–PANI samples shown in Fig. 3. Included are the coordination numbers (CN) for Fe–N and Fe–C species, and the bond lengths for the same species (*r*) and Debye–Waller factor (*σ*^2^). The values in parenthesis are the standard errors in the last digit

CN_Fe–N_	*r*_Fe–N_, Å	*σ* 2 Fe–N 10^–3^, Å^2^	CN_Fe–C_	*r*_Fe–C_, Å	*σ* 2 Fe–C 10^–3^, Å^2^
**CM**					
3.8(3)	1.98(1)	7.5(6)	1.3(2)	3.0(1)	10.3(9)

**CTRL**					
3.5(3)	1.99(1)	8.5(7)	1.3(2)	2.8(1)	9.2(8)

**MEL**					
3.8(2)	2.01(1)	7.3(6)	0.8(1)	2.7(2)	9.8(9)

**Fe–PP (Sigma-Aldrich)**					
4.0	1.93(1)	4.7(6)	8.0	3.2(1)	4.6(9)

**Fig. 4:**
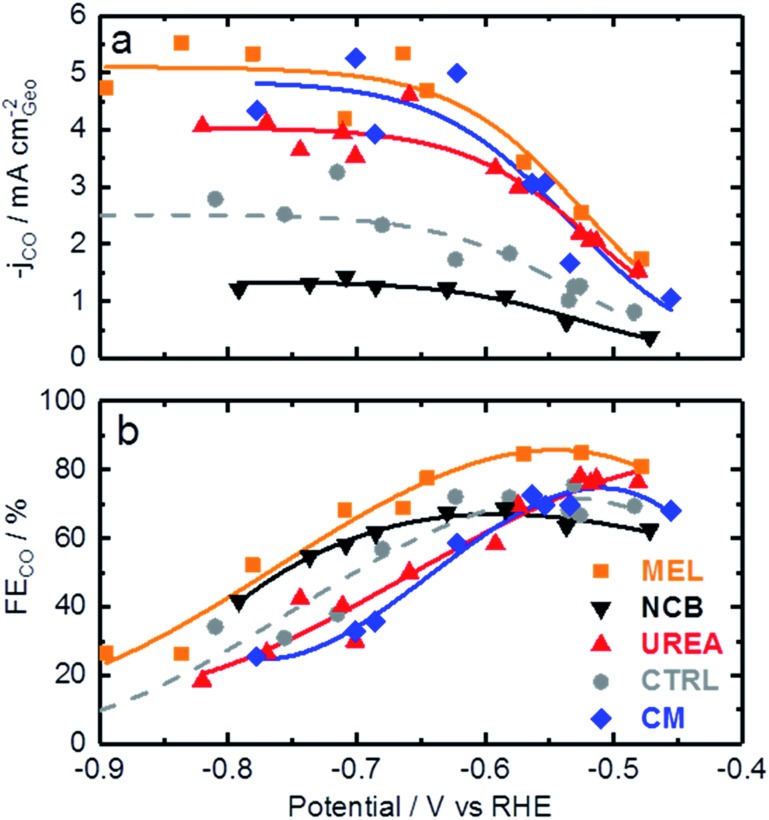
CO_2_ reduction data for various Fe–N–C catalysts based on different secondary nitrogen precursors: (a) CO generation rate, (b) faradaic efficiency towards CO production. Experimental conditions: CO_2_ saturated 0.1 M KHCO_3_, catalyst loading: 0.75 mg cm^–2^ on glassy carbon.

Now that the catalysts have been described structurally and chemically, this information can be used to better understand the catalysts electrochemical performance. Fig. 4 shows the CO production rate (a) and CO faradaic efficiency (b) of the various catalysts. From the CO production rate a kinetic region can be identified by the strong increase in performance with decreasing potential between –0.45 and –0.6 V *vs.* RHE. At lower potentials this kinetic region gives way to a plateau with maximum production rates of over 5 mA cm^–2^ for the melamine and cyanamide catalysts. The lack of potential dependence indicated by this plateau suggests that the rate limiting step has shifted to some non-electrochemical process. The faradaic efficiency towards CO production, shown in Fig. 4b, shows peak faradaic efficiencies occurring around –0.6 V *vs.* RHE with the top performing catalysts being melamine with 85% maximum CO efficiency.

**Fig. 5:**
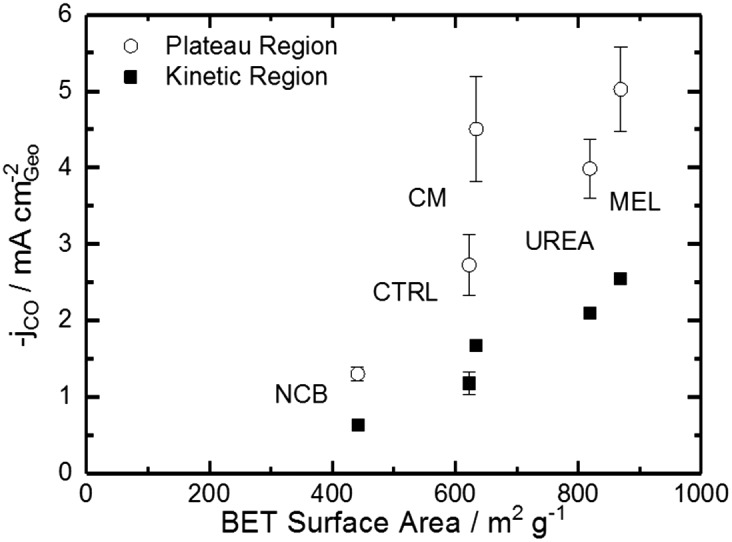
Trends of CO current density on the plateau and in the kinetic region (–0.53 V *vs.* RHE) varying with BET specific surface area.

Comparing Fig. 4 with the chemical characterizations, it is evident that the addition of the secondary nitrogen precursor has impacted both chemistry and catalyst performance. In order to understand this performance better, surface area effects must be understood. There is a strong correlation between BET surface area and performance as shown in Fig. 5. This relationship is not surprising considering the fundamental role of real surface area in heterogeneous catalysis. To reach a deeper understanding of catalysts behavior, the current can be normalized to the real surface area. This will allow a comparison of specific current densities with surface chemistries obtained from XPS results.

**Fig. 6:**
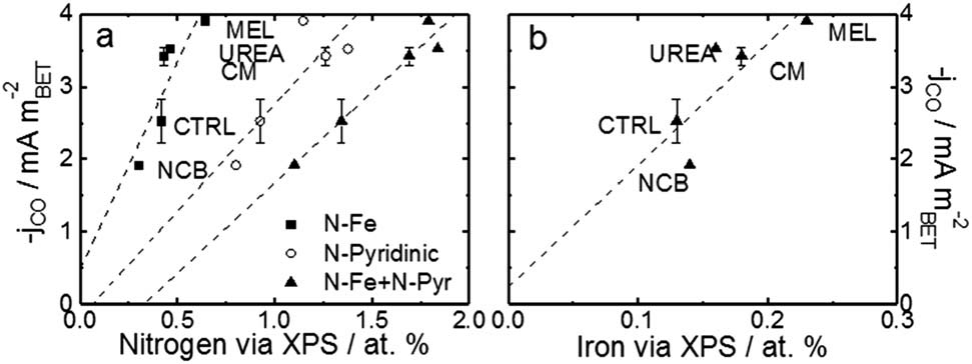
Trends of CO current densities in the kinetic region (–0.53 V *vs.* RHE) varying with (a) various pyridinic nitrogen and N–Fe and (b) surface Fe content. Current density is normalized to the specific surface area as calculated by the BET method. BET-normalized CO current densities in the kinetic region as a function of other functionalities are shown in Fig. S15 and 16.† Free energy diagrams from CO_2_ to CO over FeN_*x*_ and pyridinic-N sites are shown in Fig S17,† data which are adapted from ref. 40 and 63.

For a better understanding of the intrinsic catalytic activity of these Fe–N–C catalysts for CO_2_-to-CO conversion, specific current densities were calculated by normalizing the current in the kinetic region by the BET surface area, subsequently correlated to the chemical make-up of the surface as measured by XPS. The data show particularly good correlations with *N–Fe* species (black squares) and *pyridinic nitrogen* species (open circles) as shown in Fig. 6a. The N–Fe correlation suggests potentially active Fe–N_*x*_ sites, and the pyridinic nitrogen trend is in accordance with its catalytic properties hypothesized by Wu *et al.* (both mentioned in the Introduction).^12^,^39^ Comparing the N–Fe and N-pyridinic correlations, it can be observed that some catalysts have relatively more N–Fe (MEL and CTRL) and some relatively more N-pyridinic (UREA and CM). This observation leads to the hypothesis that both constituents contribute to the catalytic activity. For this reason, the authors also include a correlation of the specific current density with the *sum of N-pyridinic and N–Fe content* (black triangles). The linear fit of the summed data set shows a higher *R*^2^ than either of the other fits. This fit improvement suggests that both sites are likely active.

In addition to the hypothetical nitrogen active sites, literature results have also suggested that trapped iron content near the surface may be an active site for this type of catalysts.^37^,^41^,^42^ For comparison, Fig. 6b shows the correlation of specific activity with total iron from XPS. The iron has a poorer correlation than either of the nitrogen constituents, suggesting that this data does not support the hypothesis that encapsulated Fe is an active site for CO_2_RR. This uncertainty is compounded by the fact that the Fe peak is small and hard to quantify from XPS and that the samples contained not only Fe–N species, but also FeO_*x*_ species at/near the surface. The alternative theory that the metallic Fe content is a H_2_-generation site is also hard to prove.^41^,^42^ In this case, the melamine based catalyst would be expected to show the highest H_2_ faradaic efficiencies (lower CO efficiencies), but this is certainly not the case. In fact, MEL has the highest CO efficiency even though it also has the highest surface metal content.

**Fig. 7:**
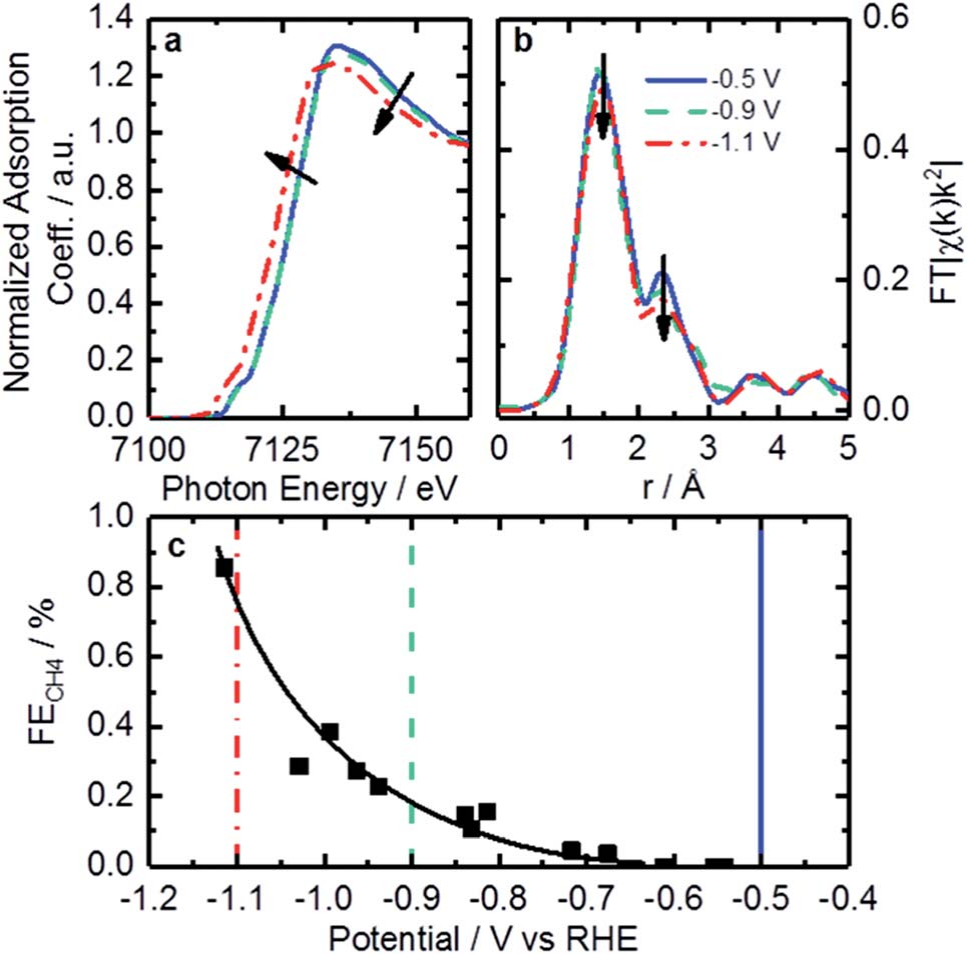
Fe K-edge XANES (a) and EXAFS (b) spectra taken under *operando* conditions in CO_2_-saturated 0.1 M KHCO_3_ at –0.5 V (solid blue curves), –0.9 V (dashed green curves) and –1.1 V (red dot-dashed curves) *vs.* RHE. (c) Faradaic efficiency to CH_4_ varying with potential. Lines are added to indicate points representative of spectra in (a) and (b).

*Operando* XAFS data were collected to elucidate possible changes in the oxidation state and local coordination of Fe. Fe K-edge XANES spectra taken under reaction conditions in CO_2_ saturated KHCO_3_ shown in Fig. 7a display a shift in edge position at –1.1 V *vs.* RHE (red trace). This shift is similar to that found on related catalysts at around +0.75 V *vs.* RHE under acidic conditions.^64^ That shift was connected to 2+/3+ active site redox behavior which had important implications concerning adsorbate bond strength.^64^ Similarly, it is likely that the shift observed in the *operando* XANES data is correlated to a 1+/2+ redox transition. This redox behavior has been observed for various iron-based macrocycles at similar potentials.^65^–^69^ These changes in active site oxidation state and coordination are also supported by changes observed in EXAFS as seen in Fig. 7b. With decreasing potential, the corresponding spectrum shows a slight decrease of both backscattering features at 1.44 Å (uncorrected), assigned to N/O, and shoulder at 2.35 Å (uncorrected), previously shown to correspond to Fe–C. Thus, the Fe–N/O coordination number decreases from 4.3 to 3.9, while the Fe–C coordination number decreases from 2.6 to 1.8 (see Tables 3 and S4† for details). The changes observed can be assigned to the reduction of surface iron oxides, detected by XPS, and increasing disorder in the material under reaction conditions.

**Table 3:** The best-fit parameters for Fe K-edge EXAFS spectra of the Fe–PANI measured under *operando* conditions are shown in Fig. 7. Included are the coordination numbers (CN) for Fe–N and Fe–C species, and the bond lengths for the same species (*r*) and Debye–Waller factor (*σ*^2^). The values in parenthesis are the standard errors in the last digit

Potential	CN_Fe–N_	*r*_Fe–N_/Å	*σ* 2 Fe–N 10^–3^, Å^2^	CN_Fe–C_	*r*_Fe–C_/Å	*σ* 2 Fe–C 10^–3^, Å^2^
–0.5 V_RHE_	4.3(8)	2.00(2)	6.8(3)	2.6(8)	3.0(2)	8.5(7)
–0.9 V_RHE_	4.2(8)	2.00(2)	7.0(4)	2.4(5)	3.0(2)	8.7(7)
–1.1 V_RHE_	3.9(9)	2.00(3)	7.2(4)	1.8(4)	3.1(2)	9.2(8)

The redox transition could have a significant impact on binding energies and reaction mechanisms. One of the interesting mechanistic questions concerning these catalysts is the role and prevalence of CO poisoning. It has been suspected that strong CO binding is one of the inhibitors of higher CO_2_RR performance as well as the cause of CH_4_ production. For that reason, the CO_2_ consumption as a function of potential was considered in the hope of seeing significant changes in catalyst behavior. This was accomplished by calculating the ratio of CH_4_ production to total CO_2_ consumed as shown in Fig. 7c. Observable in this figure is a strong increase in CH_4_ production between –0.9 and –1.1 V *vs.* RHE. The correspondence of this increase with the changes in coordination and redox behavior indicated by EXAFS suggests that the binding behavior of reactants may indeed be modified during this potential change. This observation is consistent with a proposed mechanism for photochemical methane formation on a potentially similar iron-based macrocycle catalyst.^68^ In the proposed mechanism the Fe 1+/2+ transition plays an integral role in the conversion of CO into CH_4_*via* a formyl intermediate.^68^ This theory is consistent with our observations of increased CH_4_ production, changed oxidation state, and decreased coordination. All together, we hypothesize that these FeN_*x*_ sites would provide enough room and proper binding energy for proton binding, facilitating the intermediate CHO*, which might be the rate-limiting step for CH_4_ formation. Despite the lack of systematic theoretical simulations, this mechanistic change has significant implications for future catalysts development. Specifically, for actives sites that are nitrogen-coordinated iron complexes, it is possible to adjust the iron center redox potential by adjusting ligand number/strength. This adjustment of iron center redox behavior or coordination number could be used to synthesize catalysts with higher CH_4_ yields. Conversely, it may be possible to inhibit CO-poisoning by tuning iron complexes to have lower Fe 1+/2+ redox potentials. Unfortunately, such a study is difficult on the present set of catalysts and would require a following work that looked at CO_2_RR on a set of Fe-macrocycles with varying metal center electron density of states. Such a study is outside the scope of this work.

## Conclusions

In the present work five different FeNC CO_2_RR catalysts have been explored with two goals: (1) synthesizing high performance, inexpensive CO_2_ reduction catalysts for aqueous media, (2) increasing our fundamental understanding of the active state and structure of FeNC catalysts during the CO_2_RR process. Towards the first goal, the melamine based Fe–PANI catalyst achieved a CO efficiency of 85% and a two-fold improvement in CO production rate resulting in current densities of over 5 mA cm.^–2^

Towards our second goal of increasing understanding of MNC catalysts we have three additional conclusions. Firstly, high specific surface areas are important to catalytic activity. This suggests that reaction rates are limited by adsorption and/or kinetics (*i.e.* surface events). Secondly, the comparison of specific current density with XPS data on the surface indicates that both N–Fe and pyridinic species are likely actives sites. Finally*, operando* EXAFS results indicate a reduction in metallic content between –0.9 and –1.1 V *vs.* RHE which corresponds with the redox potential of Fe 1+/2+. This event coincides with an increase in CH_4_ production, which suggests a change in active site behavior. The authors hypothesize that this change in behavior corresponds to a change in reaction mechanism that results in the onset of CH_4_ production.

## Conflicts of interest

Authors declare no conflict of interests.

## Supplementary Material

Supplementary information
