# Supplementary material for: The chemical identity, state and structure of catalytically active centers during the electrochemical CO2 reduction on porous Fe–nitrogen–carbon (Fe–N–C) materials
Source: Chem Sci. 2018 May 3;9(22):5064–73. doi: 10.1039/c8sc00491a (PMC5994794; doi:10.1039/c8sc00491a)
Supplement: Supplementary file 1 [file SC-009-C8SC00491A-s001.pdf]

Electronic Supplementary Material (ESI) for Chemical Science.
This journal is © The Royal Society of Chemistry 2018

Supporting Information

The chemical identity, state and structure of catalytically active centers during the electrochemical CO_2 reduction on porous Fe-nitrogen-carbon (Fe-N-C) materials

Nathaniel Leonard,[1] Wen Ju,[1] Ilya Sinev,[2] Julian Steinberg,[1] Fang Luo,[1] Ana Sofia Varela,[3] Beatriz Roldan Cuenya,[2,4] Peter Strasser[1*]*

1. The Electrochemical Energy, Catalysis, and Materials Science Laboratory, Department of Chemistry, Chemical Engineering Division, Technical University Berlin, Berlin, Germany
2. Department of Physics, Ruhr Universität Bochum, Bochum, Germany
3. Institute of Chemistry, National Autonomous University of Mexico, Mexico City, Mexico
4. Department of Interface Science, Fritz-Haber Institute of the Max Planck Society, Berlin, Germany

*e-mails: pstrasser@tu-berlin.de; roldan@fhi-berlin.mpg.de

Nathaniel Leonard and Wen Ju contribute equally.

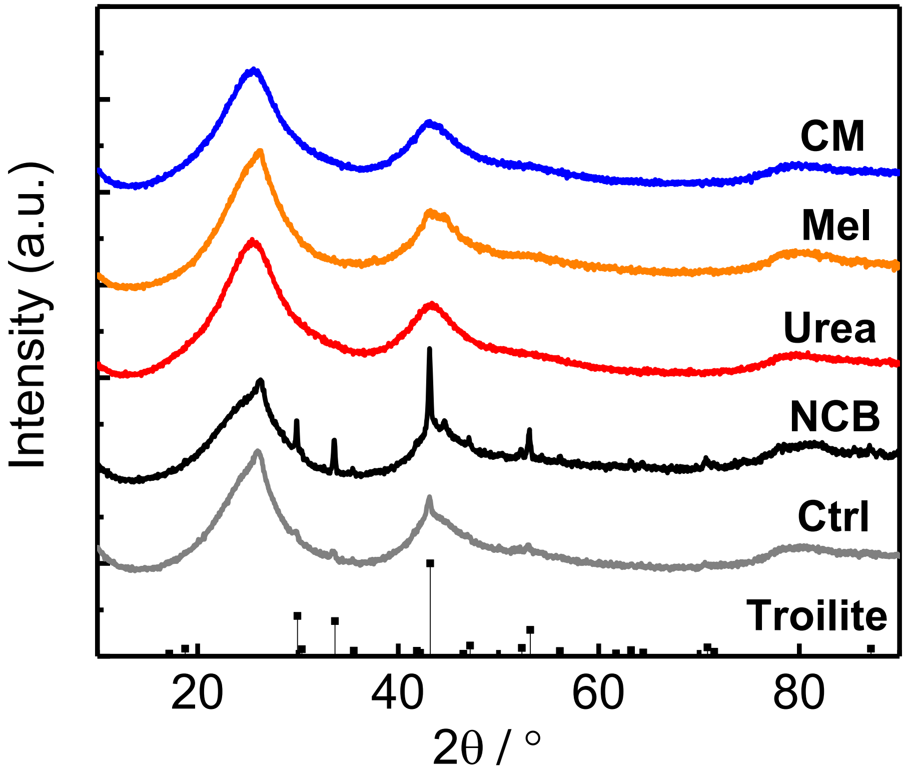

Figure S1. Powder X-ray diffraction of catalysts from this project. At the bottom a diffraction pattern of troilite from Skala et al[1] is displayed.

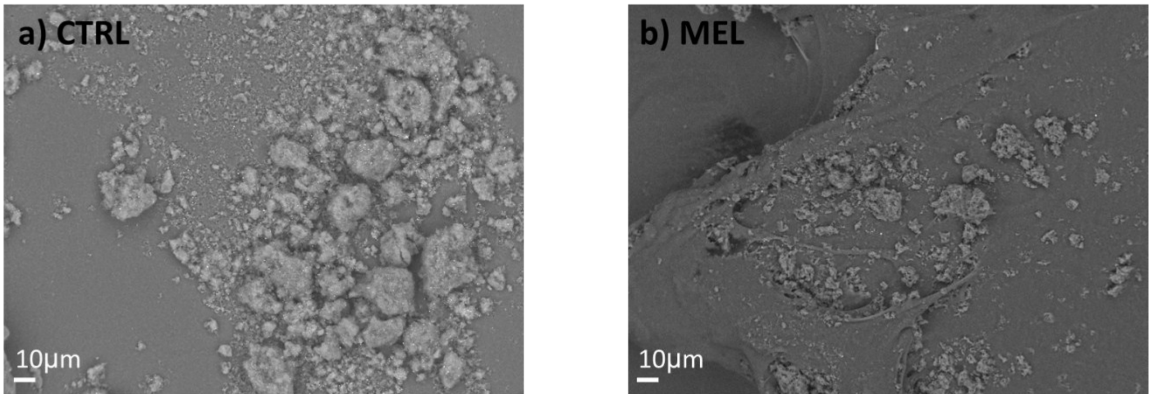

Figure S2. Typical Scanning Electron Microscopy images of a) CTRL and b) MEL PANI-Fe electrocatalysts.

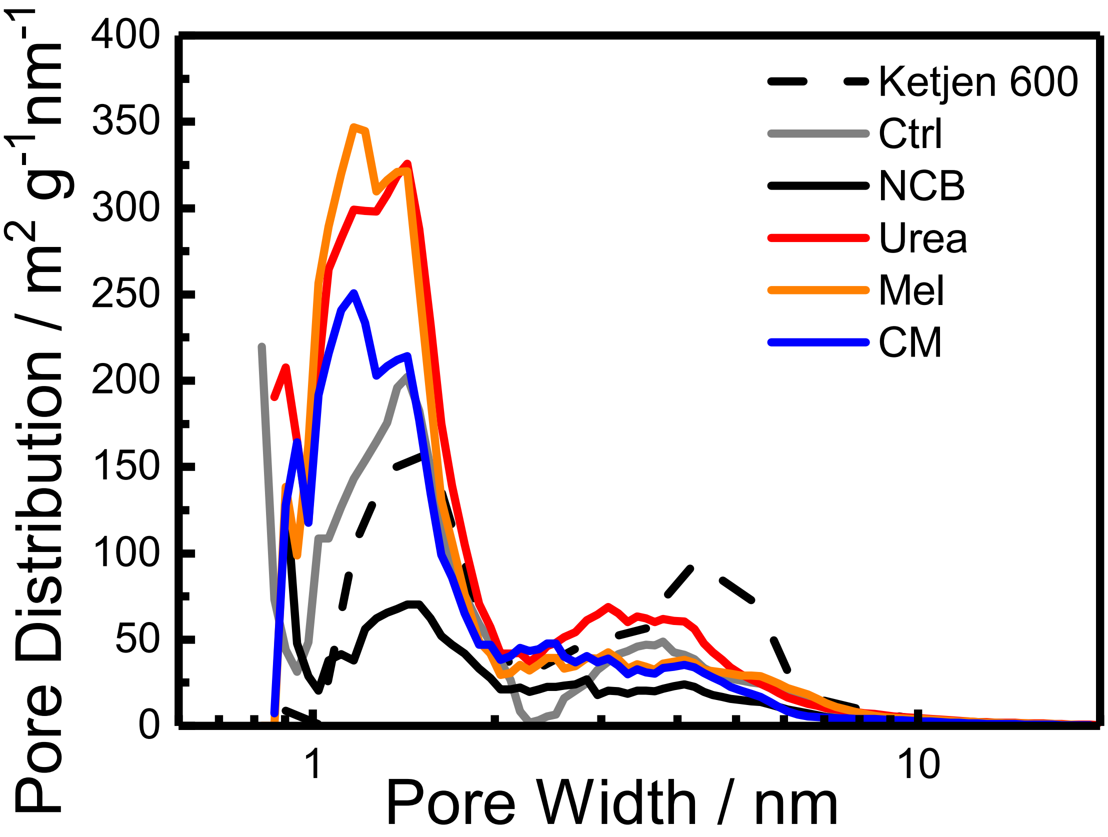

Figure S3. Pore size distribution of support and catalysts.

Area ($Fe-N_x$) = BET * N-Fe % Equation SI 1

BET: N_2 physiosorption BET surface area / $m^2\ g^{-1}$

N-Fe %: XPS surface Mole fraction of Fe coordinated Nitrogen

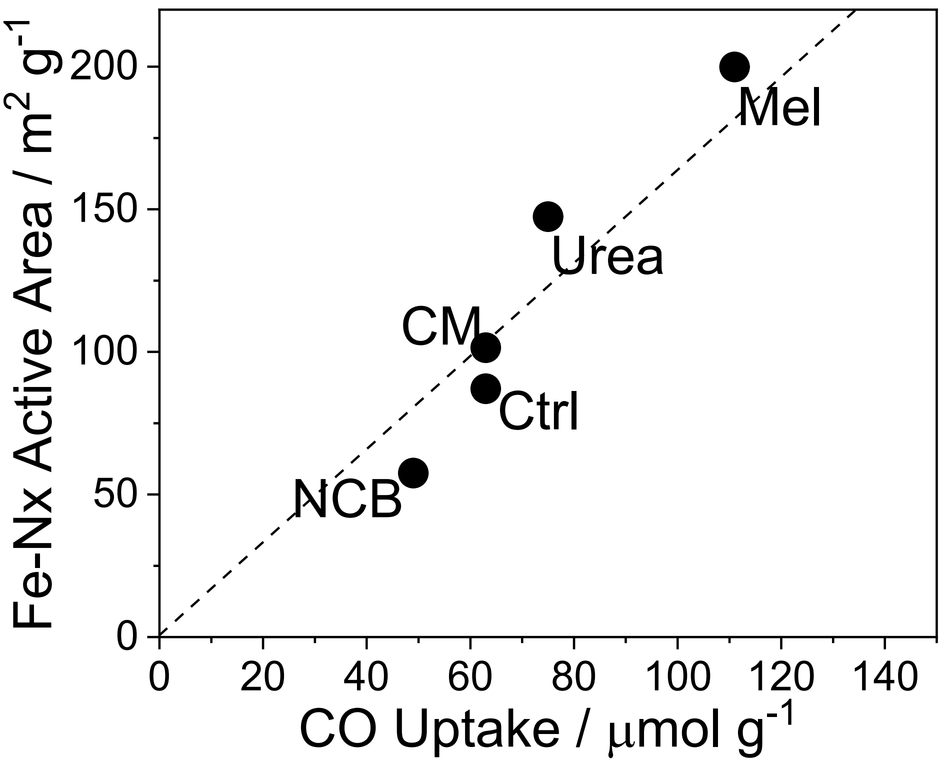

Figure S4. Combination of CO-Chemisorption measurements and interfacial Fe-Nx sites area.

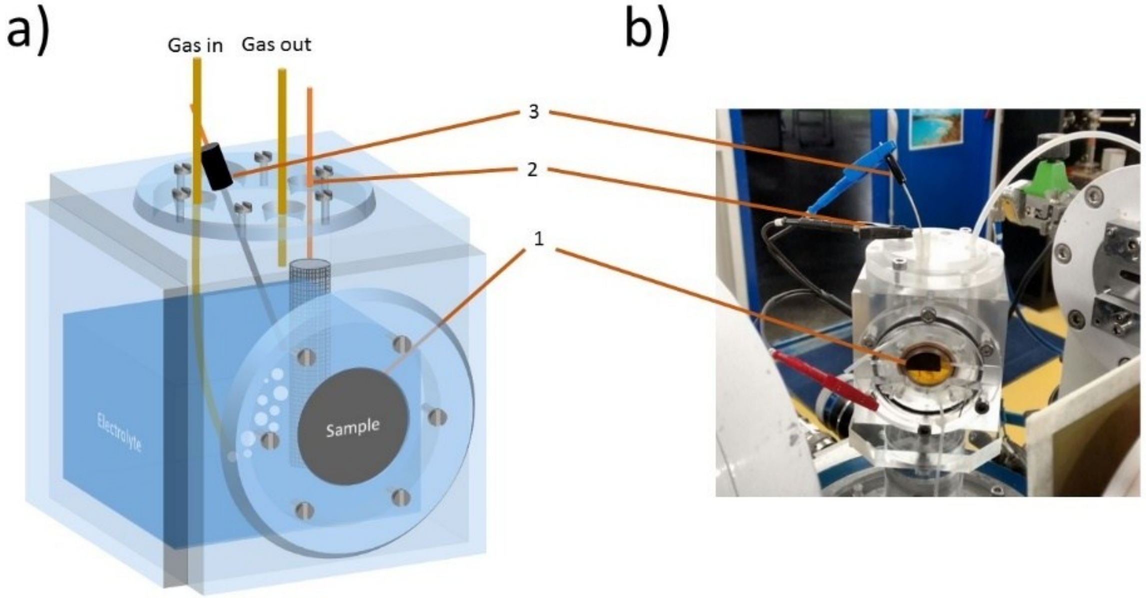

Figure S5. Operando XAS cell used in this work: a) schematic illustration of the cell, 1 – working electrode (GDE with the ink sample) sealed with Kapton tape, 2 – Pt gauze counter electrode, 3 – leak-free Ag/AgCl reference electrode. b) The cell during measurements at SAMBA beamline of SOLEIL synchrotron light source (Paris, France).

Table S1. Characteristics of Secondary Nitrogen Precursors Used in this Work

Nitrogen Precursor	***Formula***	***MW / g mol^{-1}***	***N/C***
Cyanamide	CH_2N_2	42	2/1
Melamine	$C_3H_6N_6$	126	6/3
Urea	CH_4N_2O	60	2/1
Nicarbazin	$C_{19}H_{18}N_6O_6$	426	6/19

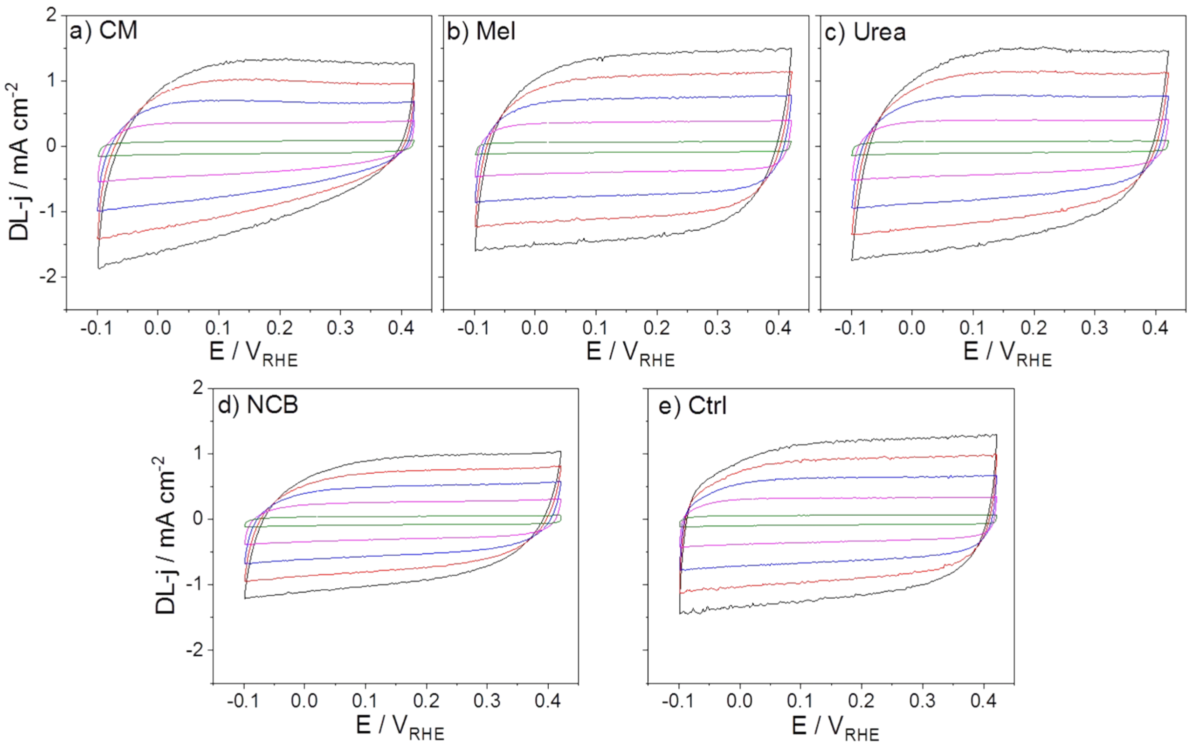

Figure S6. Cyclic voltammetry of the five Fe-N-C catalysts conducted in CO_2-saturated 0.1 M $KHCO_3$ at various scan rates for estimation of the double layer (DL) capacity. a) CM, b) Mel, c) Urea, d) NCB and e) Ctrl. Cycle voltammetry was performed between -0.1 and 0.42 V vs. RHE to avoid the interference of the faradaic process and at the scan rate were 20 mV s^{-1}, 15 mV s^{-1}, 10 mV s^{-1}, 5 mV s^{-1}, 1 mV s^{-1}. Double layer current densities are utilized to determine the double layer capacitance, which is proportional to the double-layer interfacial area. Catalysts loading: 0.75 mg cm^{-2}.

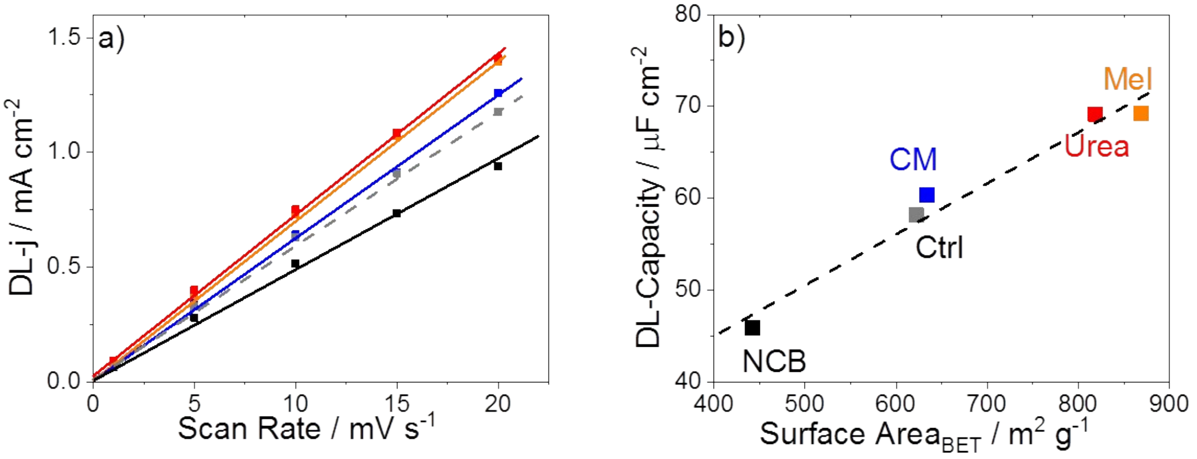

Figure S7. a) Double layer current densities on 5 different Fe-N-C catalysts as a function electrode potential scan rate to extract the double layer capacity; b) Correlation of double layer capacity and the N_2 adsorption derived BET surface area.

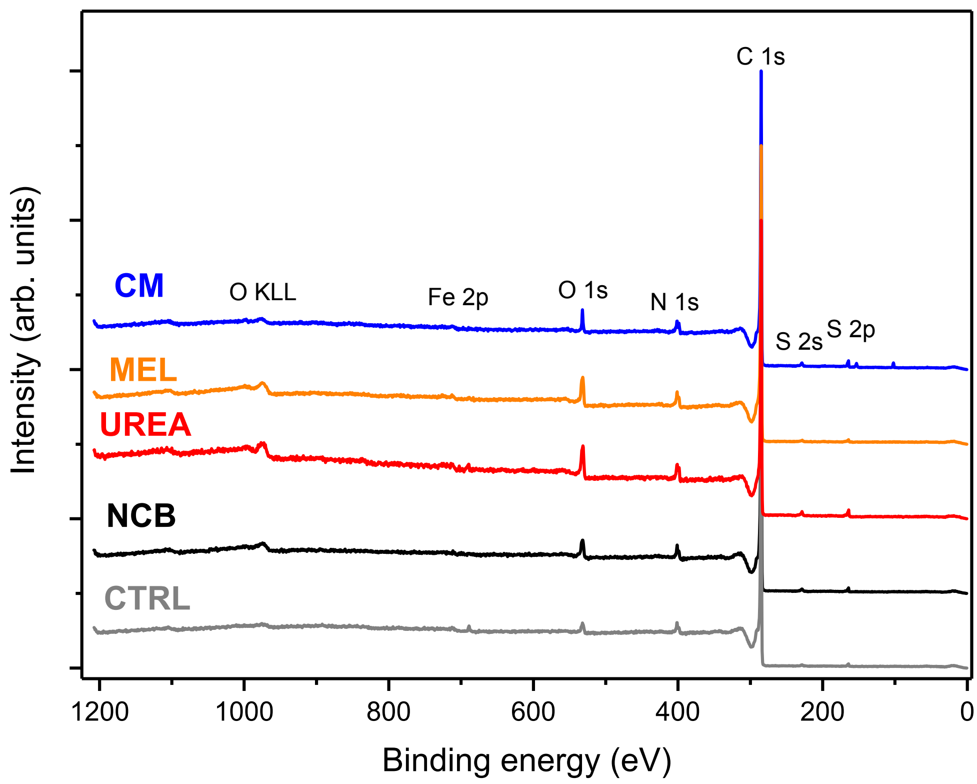

Figure S8. Survey XPS spectra of Fe-PANI materials with the main features assigned.

Table S2. Sample composition as seen by XPS

sample	*C*	*N*	*O*	*S*	*Fe*
NCB	94.8	2.9	1.7	0.5	0.1
CTRL	93.3	3.3	3.1	0.2	0.1
CM	93.1	3.9	1.6	1.3	0.2
MEL	90.9	3.6	4.8	0.5	0.2
UREA	92.8	3.7	2.0	1.3	0.2

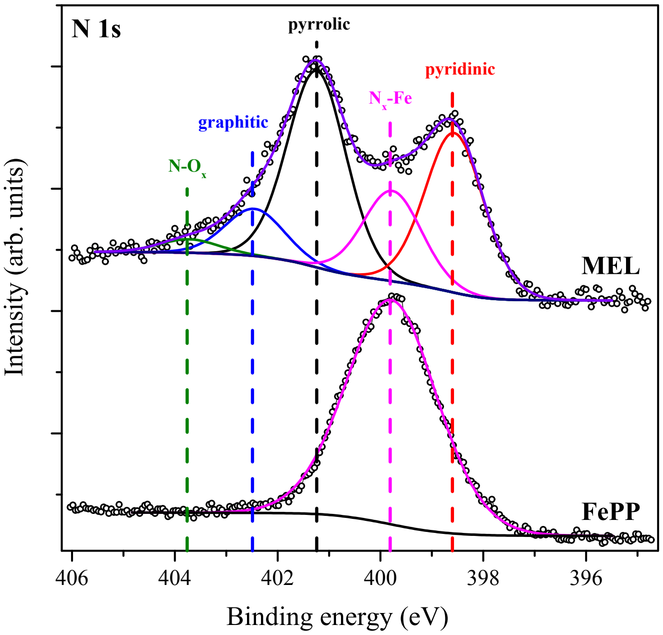

Figure S9. High resolution N 1s XPS spectra of a PANI-Mel sample and commercial Fe Protoporphyrin.

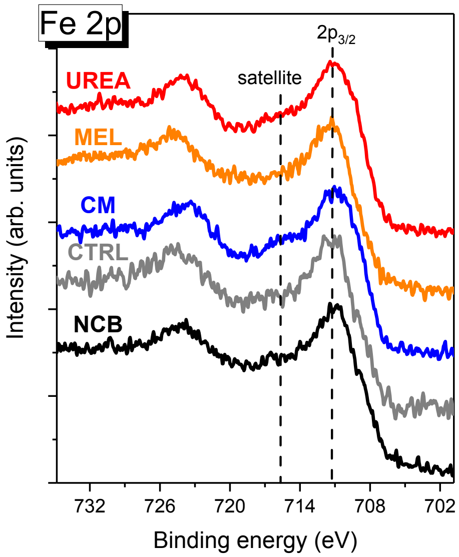

Figure S10. Fe 2p high resolution XPS spectra of Fe-PANI catalysts.

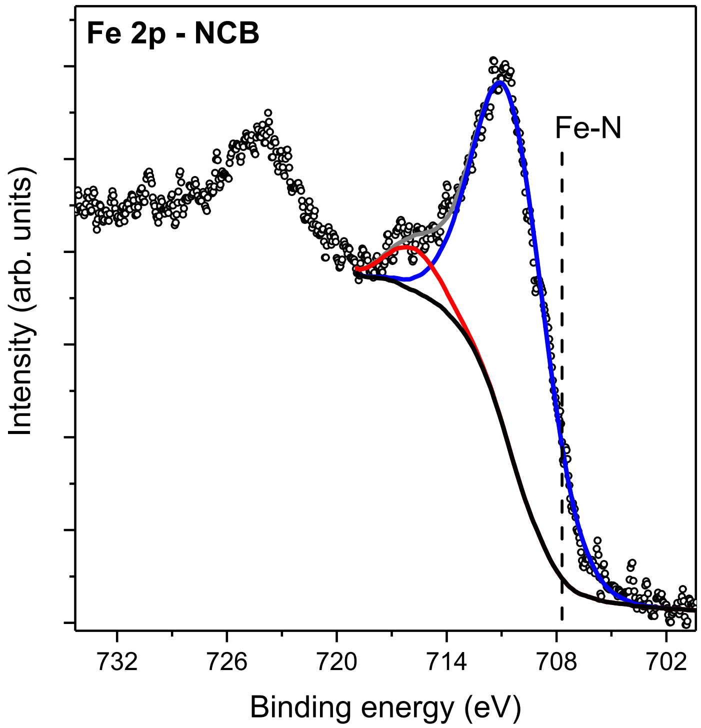

Figure S11. Deconvolution of Fe $2p_{3/2}$ XPS spectrum representative of the NCB sample. Open dots show experimental data, solid blue line – main Fe $2p_{3/2}$ peak, solid red line – shake up satellite. Vertical dashed line shows position of $Fe2p_{3/2}$ reported for Fe-porphyrin.

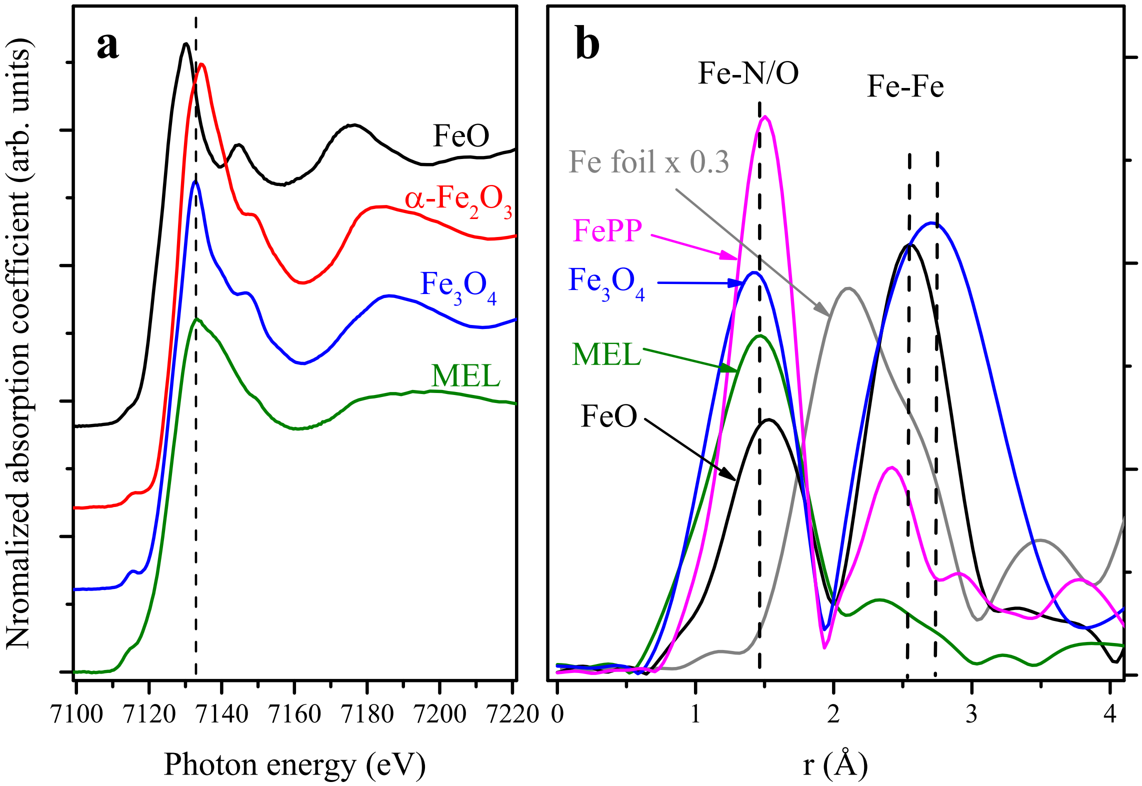

Figure S12. Fe K-edge XANES (a) and EXAFS (b) spectra of Fe-PANI MEL sample, iron foil, commercial iron protoporphyrin (FePP, Sigma Aldrich) and the most common iron oxides. The intensity of iron foil spectrum in (b) is reduced by factor of 3 for better display.

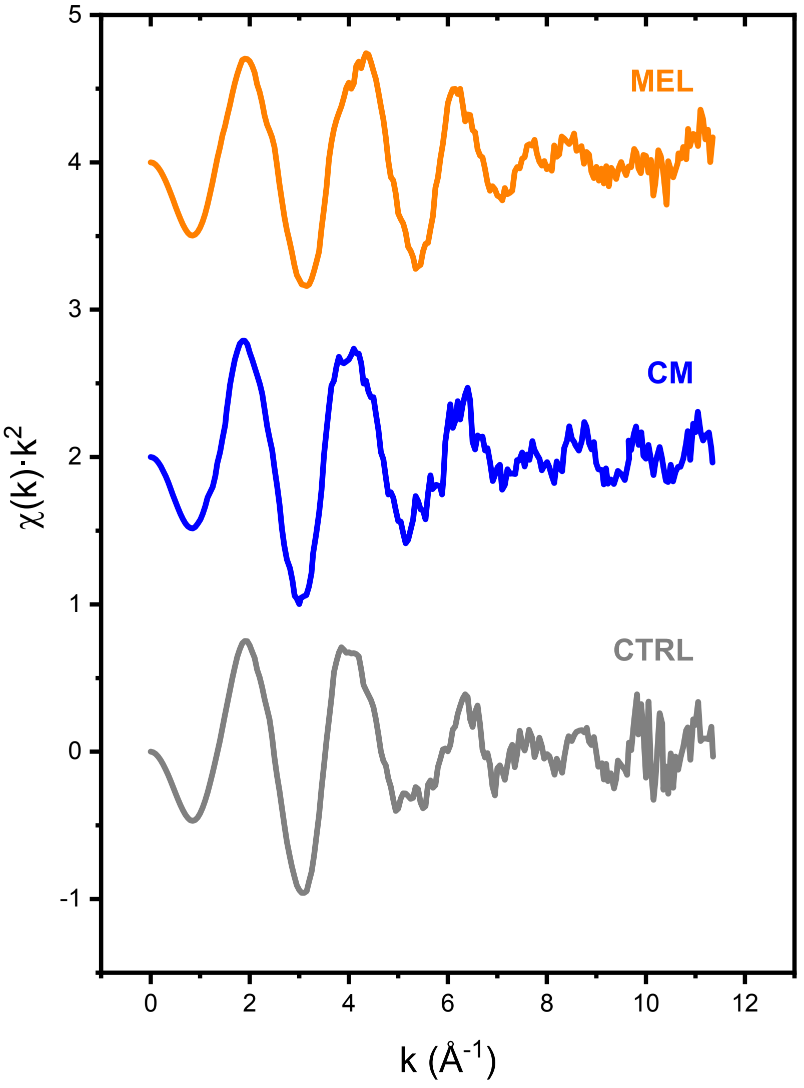

Figure S13. Fe K-edge k2-weighted EXAFS data of Fe-PANI samples in k-space.

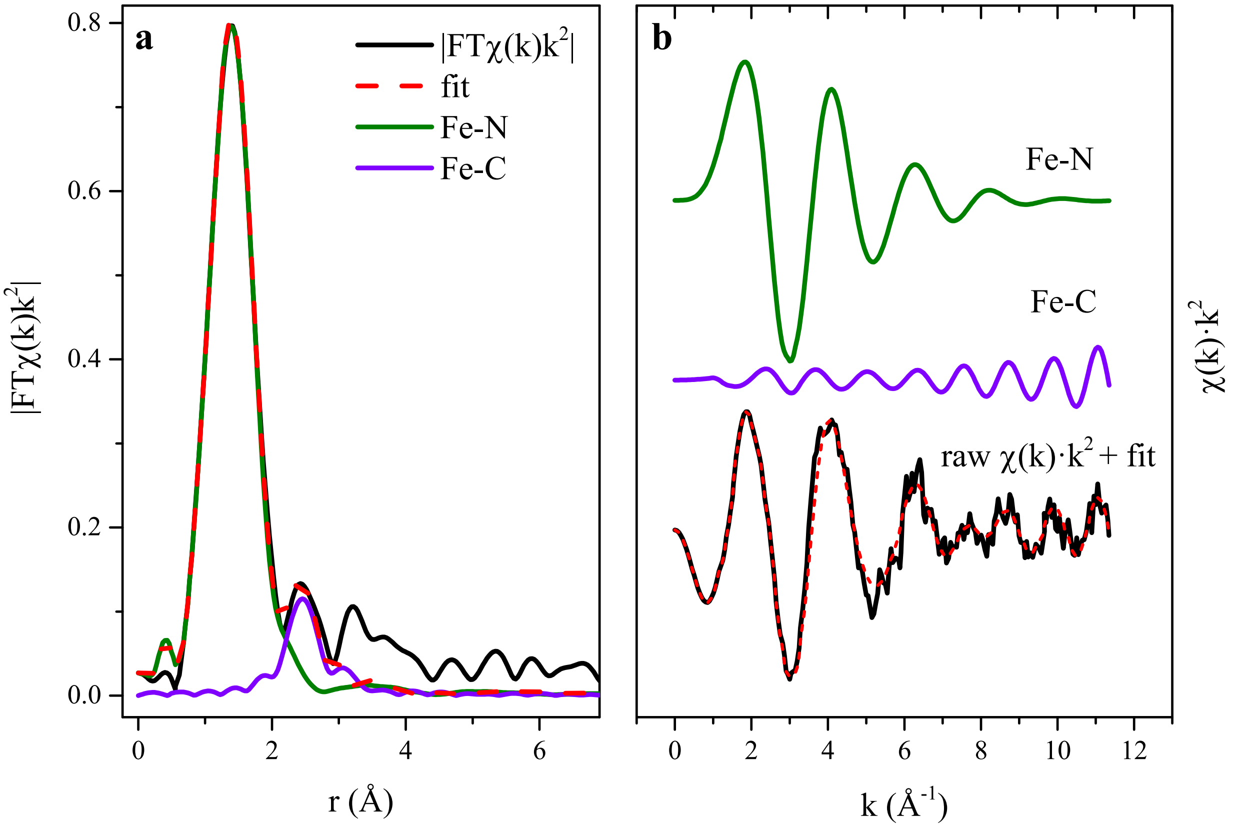

Figure S14. FeK-edge EXAFS analysis as exemplified by CM sample. Panel **a**: the fit in the Fourier Transformed space, solid black line - raw data, dashed red line - fitting model. Panel **b** shows the corresponding curves in the k-space along with the Fe-N and Fe-N single scattering components.

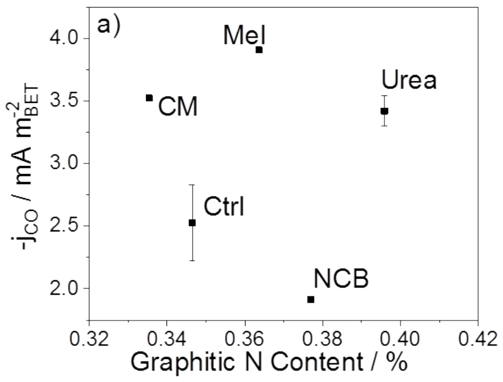

Figure S15. BET normalized CO partial current density as a function of a) Graphitic N content and b) Pyrrolic N content.

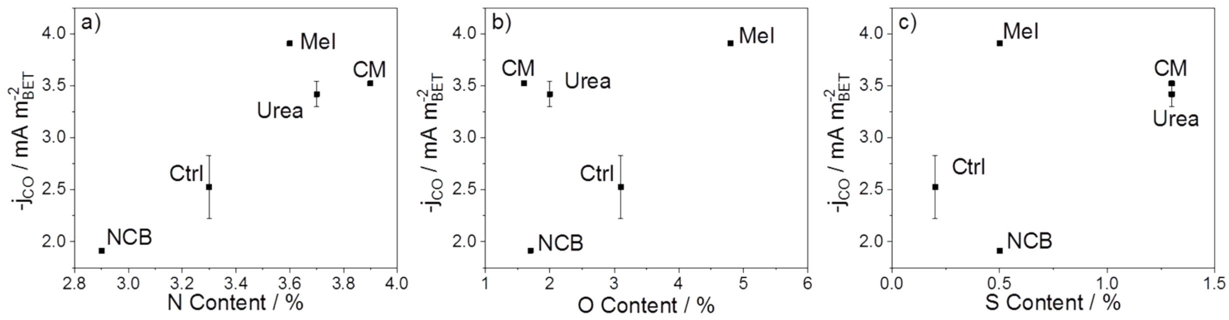

Figure S16. BET-normalized CO partial current density as a function of a) Nitrogen content, b) Oxygen content and c) Sulfate Content.

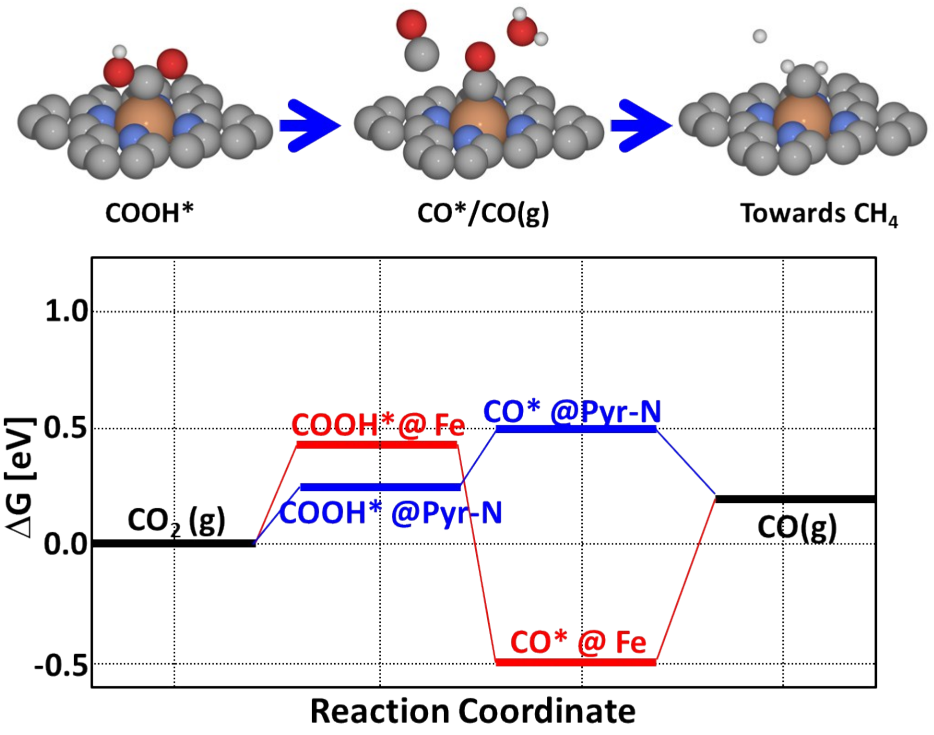

Figure S17. Reaction mechanism and DFT free energy diagram of CO2RR to CO on Fe-Nx site and pyridinic nitrogen. (Free Energy data are adapted from [[2]] and [[3]])

References:

1 Skála, R., Císařová, I. & Drábek, M. in *American Mineralogist* Vol. 91 917 (2006).

2 Wu, J. *et al.* Achieving Highly Efficient, Selective, and Stable CO2 Reduction on Nitrogen-Doped Carbon Nanotubes. *ACS Nano* **9**, 5364-5371, doi:10.1021/acsnano.5b01079 (2015).

3 Ju, W. *et al.* Understanding activity and selectivity of metal-nitrogen-doped carbon catalysts for electrochemical reduction of CO2. *Nature Communications* **8**, 944, doi:10.1038/s41467-017-01035-z (2017).
